# How ovarian hormones influence the behavioral activation and inhibition system through the dopamine pathway

**DOI:** 10.1371/journal.pone.0237032

**Published:** 2020-08-13

**Authors:** Jia-Xi Wang, Jin-Ying Zhuang, Lulu Fu, Qin Lei, Mingxia Fan, Weidong Zhang

**Affiliations:** 1 School of Psychology and Cognitive Science, East China Normal University, Shanghai, China; 2 Department of Physics, Shanghai Key Laboratory of Magnetic Resonance, East China Normal University, Shanghai, China; UiT Norges arktiske universitet, NORWAY

## Abstract

The behavioral activation system (BAS) and the behavioral inhibition system (BIS) have been proposed to relate to stable traits that predict inter-individual differences in motivation. Prior reports point dopamine (DA) pathways, mainly including ventral tegmental area (VTA) and substantia nigra (SN), implicate in subserving reward-related functions associated with BAS and inhibitory functions related with BIS. However, as an important factor that affects DA releasing, it remains an open question whether the ovarian hormones may also be related to BIS/BAS. Here, to investigate effects of the estradiol (E2) and progesterone (PROG) on BIS/BAS and related DA pathways, we employed a BIS/BAS scale and the resting-state functional magnetic resonance imaging (fMRI) during the late follicular phase (FP) and the mid-luteal phase (LP). On the behavioral level, when women had high PROG levels, their E2 levels were found positively correlated with BIS scores, but those women whose PROG levels were low, their E2 levels were negative correlation with BIS scores. On the neural level, we demonstrated BAS was related with the VTA pathway, included brain reward regions of nucleus accumbens (NAc) and orbitofrontal cortex (OFC). Meanwhile, the BIS was correlated with the SN-dorsolateral prefrontal cortex (dlPFC) pathway. ROI-based resting-state functional connectivity (RSFC) analyses further revealed that, RSFC between the SN and dlPFC was modulated by ovarian hormones. With higher PROG levels, increased E2 levels among women were accompanied by stronger RSFC of the SN-dlPFC, but when PROG levels were low, E2 levels were negatively correlated with the SN-dlPFC RSFC. These findings revealed a combined enhancement effect of E2 and PROG on BIS, and the SN-dlPFC pathway was mainly involved in this process.

## Introduction

Over the past decade, one major challenge in human cognitive neuroscience is to understand how specific differences in brain physiology relate to global psychological phenomena such as personality traits. Clinical researches on pathological deviations, such as impulsivity and addiction, have provided a possible relationship between the personality traits of inhibitory control on reward-seeking and neurological disorder [1]. For example, psychopathy is a personality disorder with strong link to impulsive action, cerebrum morphological findings indicate decreased prefrontal cortex gray matter volume in psychopathy [2]. Likewise, psychopaths show stronger activity in the nucleus accumbens (NAc) during rewarding-seeking task and weaker intrinsic functional connectivity between NAc and prefrontal cortex [3].

Recently, an increasing number of studies in healthy samples have also linked individual differences in self-control on rewards to neural nodes including ventral midbrain, basal ganglia, and prefrontal cortex [4]. A few researches in healthy adults associated the specific self-reported personality traits with resting-state functional connectivity (RSFC). The RSFC measures the degree of correlated neural activity between different brain regions while the brain is at rest and is thought to reflect the strength of functional connections. In one study, researchers found greater RSFC between substantia nigra (SN) and thalamus was related to higher self-reported impulsivity [5]. Here, we would like to concern with a personality traits associated both impulsivity and inhibition motivation, and explore the related RSFC. Based on findings from animal learning paradigms, these two dimensions of personality could be reflected by two aspects of nervous system, the behavioral activation system (BAS) and the behavioral inhibition system (BIS) [6]. In Gray’s theory, the BAS responds to conditioned stimuli of reward and non-punishment, elicits positive emotions, and leads to approach behavior and active avoidance. The BIS responds to conditioned stimuli of punishment and non-reward, as well as to novel stimuli and innate fear stimuli, it elicits the affective state of anxiety, and leads to behavioral inhibition [7]. The BIS/BAS scales were popular validated in prior personality studies, which the BAS correlates strongly with impulsivity and rewards, while the BIS relates to the inhibition and punishment [8].

Neurophysiological findings further indicate that BAS correlated with reward-related blood oxygen level-dependent (BOLD) activation, implicating dopamine (DA) pathways involving the ventral tegmental area (VTA) to NAc and orbitofrontal cortex (OFC) [9]. The NAc and OFC widely participant in experiments that using rewards and appetizing stimuli, meanwhile, DA, which projected from the VTA, is seen as the necessary for motivation to acquire food, money or addictive drug [10]. The neural basis of BIS is less clearly specified than that of BAS, but DA pathways are also believed critically to the BIS [11]. Some researchers suggest that BIS corresponds to a withdrawal system. The withdrawal system is activated by aversive stimulation, the neuroanatomical basis of this system is supposed to be the right dorsolateral prefrontal cortex (dlPFC), the right temporal polar region, and the basal ganglia [12]. The basal ganglia, including the VTA and SN, is an important DA transfer node in DA pathways [13]. Unlike the VTA-NAc projection pathway, the SN pathway has more interaction with the prefrontal cortex and is responsible for the reward-related cognition and involves in the initiation and movement of muscle [14]. Thus, as the BIS need more inhibition response which is controlled by the prefrontal cortex, maybe the BIS is more related to the SN pathway compared with the BAS which more correlated to the VTA pathway.

On the other side, ovarian hormones, such as estradiol (E2) and progesterone (PROG), which regularly fluctuate during women’s menstrual cycle, are reported to have power to influence the neuroplasticity and activation of specific DA pathway in the adult brain [15, 16]. Generally, the menstrual cycle can be divided into two main phases: the follicular phase (FP), between onset of menses and ovulation with rising levels of E2 and very low levels of PROG; the luteal phase (LP), starting after ovulation until the onset of the next menses characterized by high levels of PROG and the second rising levels of E2 in the mid-LP [17]. Using the ^18^F-fluoroclebopride (FCP) and a high-resolution micro positron emission tomography (PET) scanner during the FP and LP of the menstrual cycle in female cynomolgus monkeys, the ratios of DA receptor availability were significantly higher during the LP compared to the FP in the dorsal striatum which belonged to the SN pathway [18]. Neuroimaging research also revealed that both monetary rewards and high-calorie-rewarding foods (e.g., cakes, cookies, ice cream) could induce greater activity in the VTA pathway during the FP than that during the LP [19, 20].

In addition, several studies have associated women’s some personality traits to ovarian hormones. Anxiety traits were found to have a positive correlation with PROG levels among different women [21]. Women in the mid-LP were also reported to have a negative emotion bias in both behavioral and neural studies [22]. Conversely, the impulsivity often reported positively correlated with E2 levels, but have a negative correlation with PROG. In animal’s studies, for example, E2 administration to ovariectomized female rats increased their impulsivity to get cocaine [23]. However, when E2 and PROG were given together, PROG diminished the facilitative effects of E2 [24]. Similar results were found in humans. E2 levels were positively correlated with the magnitude of subjective (e.g., feeling “high”) and physiological (e.g., heart rate) stimulation of *d*-amphetamine when PROG levels were low during the FP. But during the LP, these effects of E2 were disappeared when PROG was high [25, 26].

Taken together, in the present study, we aimed to investigate how E2 and PROG affected the BIS/BAS through DA pathways. Considering in previous studies, different levels of PROG could regulate E2’s effect on the impulsivity to the reward stimuli, we would like to pick two menstrual phases which had different PROG levels but similar E2 levels to explore the counter effect between PROG and E2 on BIS/BAS. Therefore, women were instructed to perform BIS/BAS scales in either the late FP (high E2, low PROG) or the mid-LP (high E2, high PROG). Then, all the participants finished a resting-state functional magnetic resonance imaging (fMRI) scanning with their eyes open but did nothing during the scanning. The concentration of E2 and PROG would be collected in different menstrual phases, the RSFC would be used to analyze neural circuit dynamics including the VTA and SN pathways.

We hypothesized that, firstly, the BIS/BAS would relate to DA pathways, specifically, the BAS would have more relationship to the VTA pathway while the BIS would correlate with the SN pathway. Secondly, when PROG was low, E2 would positively correlate with the BAS and have a negative correlation with BIS, but these correlations would reverse when PROG levels were high. Thirdly, we expected these effects would also show up in the menstrual group analyses between the late FP and mid-LP, with the late FP group had higher BAS scores and enhanced VTA pathway, but the mid-LP had higher BIS scores and enhanced SN pathway.

## Methods

### Participants

This study enrolled 53 healthy, heterosexual, right-handed, female undergraduates [19–28 years old, mean age = 22.77 years, standard deviation (SD) = 2.35]. The participants were recruited from a larger, common subject pool with certain inclusion/exclusion criteria, only individuals who were heterosexual, reported having a 28–30-day menstrual cycle, and did not use any form of hormones in the previous 3 months were included.

We used the backward counting method to predict each participant’s late FP, mid-LP, and next menstrual onset. This method has been successfully used to predict other effects of theoretical interest [27–29]. The late FP included the period of 14 to 16 days prior to a woman’s next predicted menstrual onset, and the mid-LP included the period of 6 to 8 days prior to her next predicted menstrual onset. 28 women in the late FP with a mean age of 22.54 years (SD = 2.18) and 25 women in their mid-LP with a mean age of 23.04 years (SD = 2.54) were subjected to the resting-state fMRI. The testing order was randomized across participants and phases.

All participants had normal or corrected-to-normal vision. No participants reported a history of a psychiatric disorder or current use of psychoactive medications. The protocol was reviewed and approved by the University Committee on Human Research Protection (UCHRP) of East China Normal University (approval letter of UCHRP: HR 096–2018); and the study was conducted in accordance with the Declaration of Helsinki. Written informed consent was obtained from all participants, and they were compensated 50 RMB per hour.

### Hormone assays

A saliva sample was obtained from each participant immediately before scanning. To control for circadian influences on hormone level, all experimental sessions were performed between 12:00 pm and 7:00 pm [30]. Each participant drooled ~2mL of saliva passively into a collection, and each saliva sample was preserved in a refrigerator (−20°C). All samples were processed for E2 and PROG levels with DRG International ELISA kits and the ELISA results were measured with a Thermo Devices Multiskan MK3 by ThermoFisher Scientific Shanghai Company. Independent *t*-sample tests were conducted on each hormone separately to verify cycle phases. Correlation analysis was further done between E2 and PROG.

### Positive affect and negative affect schedule

After finishing the saliva sample collection, participants completed the Positive Affect and Negative Affect schedule (PANAS) [31]. They were asked to indicate how they felt right then, at the present moment. Participants rated each emotion [10 positive affect (PA) items and 10 negative affect (NA) items)] on a 5-point unipolar response scale, where 1 = *very slightly or not at all*, 2 = *a little*, 3 = *moderately*, 4 = *quite a bit*, and 5 = *extremely*. We computed means (*M*) of 10 items separately for PA and NA. Independent *t*-tests were done to make sure women in the late FP and the mid-LP had similar emotional state. The *M* values were reported with their *SD*.

### Behavioral inhibition and activation scale

Before the resting-state scanning, participants completed a Chinese version of the BIS/BAS Scale [8]. The BIS subscale relates to the anticipation of a punishment and avoidance of cues to negative outcomes (5 items, e.g., ‘I worry about making mistakes’). The BAS is divided into three subscales: a positive response to the occurrence or anticipation of reward (reward responsiveness, 4 items, e.g., ‘When I get something I want, I feel excited and energized’), a persistent pursuit of desired goals (drive, 4 items, e.g., ‘When I want something, I go all-out to get it’), and a desire for new rewards and willingness to approach a potentially rewarding event on the spur of the moment (fun-seeking, 5 items, e.g., ‘I crave excitement and new sensations’). Responses were made on a 4-point scale, with 1 = *strong disagreement*, 2 = *slight disagreement*, 3 = *slight agreement*, 4 = *strong agreement*. Sum of each items was calculated as scores for the four subscales. A higher score indicates a greater level of that particular trait on each subscale.

Independent *t*-tests on each subscale were done to analyze differences between the late FP and mid-LP groups. Pearson’s correlations were used to examine the direction of relationship between each subscale and ovarian hormones across whole samples with ignoring menstrual groups. Further, we ran moderating effect analyses to determine whether the magnitude of change in PROG between individuals could inverse the relationship between E2 and BIS/BAS. Moderating effect was tested using Model 1 in PROCESS for SPSS [32], which tested moderating effect via calculation of 5000 bias-corrected bootstrap 95% confidence intervals (CI). Significance for all analyses providing *p*-values were set at *p* < .05.

### Image acquisition

The resting-state fMRI scanning was conducted on a 3-T Siemens scanner at the Functional MRI Lab. Functional images were acquired with a gradient echo-planar imaging sequence, 2000-ms repetition time (TR), 30-ms echo time (TE), 384-mm field of view (FOV), 3 mm × 3 mm × 3.5 mm voxel size and 33 slices. The images associated with the first ten TRs were discarded to allow for T1 equilibration. Participants were instructed to relax with their eyes open during the resting-state scanning, which lasted about 8 minute. Prior to fMRI scanning, a high-resolution structural image was acquired with a T1-weighted, multiplanar reconstruction sequence (TR = 2530 ms, TE = 2.98 ms, FOV = 256 mm, 1 mm × 1 mm × 1 mm voxel size, and 192 slices).

### Preprocessing

Preprocessing was performed in DPABI module V3.2. All volume slices were corrected for different signal acquisition times. Then, the time series of images for each participant were realigned. Individual structural images were co-registered to the mean functional image after realignment. The transformed structural images were then segmented into gray matter (GM), white matter (WM) and cerebrospinal fluid (CSF) [33]. To remove the nuisance signals, the Friston 24-parameter model was utilized to regress out head motion effects from the realigned data. Regression with autoregressive models of motion incorporating 6 head motion parameters, 6 head motion parameters one time point before, and the 12 corresponding squared items were corrected at the individual level [34, 35]. The signals from WM and CSF were regressed out to reduce respiratory and cardiac effects. In addition, linear and quadratic trends were also included as regressors since the BOLD signal exhibits low-frequency drifts. Spatial smoothing (4-mm full-width at half-maximum kernel) were applied to the functional images. Further, temporal filtering (0.001–0.08 Hz) was then performed on the time series.

### Whole brain resting-state functional connectivity analysis

RSFC analysis was continue to implement in DPABI based on a series of regions of interest (ROI). The ROI-to-whole-brain RSFC analysis allowed us to test hypotheses regarding the relationship between ovarian hormones and DA pathways. A ROI with a radius of 6mm containing 28 voxels centered in midline (MNI coordinated: 0, -15, -12) was selected to represent the location of VTA, and 2 ROIs with a radius of 6mm containing 28 voxels (1 for the left and 1 for the right hemisphere) centered in the SN (MNI: ±12, -12, -12) to represent the location of bilateral SN [36]. Then, the average connectivity strength was extracted from each participant’s preprocessed data to reveal the ROI-to-whole-brain RSFC maps with respect to VTA and SN. RSFC maps corresponding to VTA and SN were respectively included in an Independent *t*-test with two group (late FP, and mid-LP). Moreover, for each RSFC map, a series of separate whole-brain voxel-wise regression analysis were conducted for BIS/BAS subscales, levels of PROG, and E2, which ignoring all participants’ menstrual group. Covariate of age was used in these analyses in order to control for the confounding effects of age.

All analyses were completed in SPM8. The results were reported at a family-wise error (FWE) threshold *p* < 0.05, corrected for multiple comparisons at the cluster level. Activations were localized with the AAL template in Micron [37].

### Region-of-interest resting-state functional connectivity analyses

The BIS/BAS subscales relevant clusters were further evaluated with ROI-to-ROI RSFC analyses to identify potential interaction between hormones and BIS/BAS on DA pathways. The average connectivity strength was extracted from individual RSFC maps in ROIs with radius of 6mm located in regions of the OFC and dlPFC that demonstrated strong connectivity with VTA and SN. Pearson correlations and moderating effects analyses were conducted between these ROI-to-ROI RSFC and ovarian hormones levels in SPSS 19.0.

## Results

### Demographics

Two participants were excluded for excessive movement during scanning (≥2.5-mm maximum displacement in the x, y or z dimension; and/or ≥ 2.5 ° angular motion). Two participants were excluding for having longer menstrual cycle than 30 days after we tracked them to their next menstrual cycle. Of the 49 remaining participants (mean age = 22.86 years, *SD* = 2.29; range: 19–28), 25 participants were scanned during their late FP (mean age = 22.52 years, *SD* = 2.38), and 24 participants were scanned during their mid-LP (mean age = 23.21 years, *SD* = 2.19). Age did not differ significantly between the two menstrual phase groups (*t*_2,47_ = 1.052, *p* = 0.297, Cohen’s *d* = 0.307).

### Hormone assays

**Fig 1:**
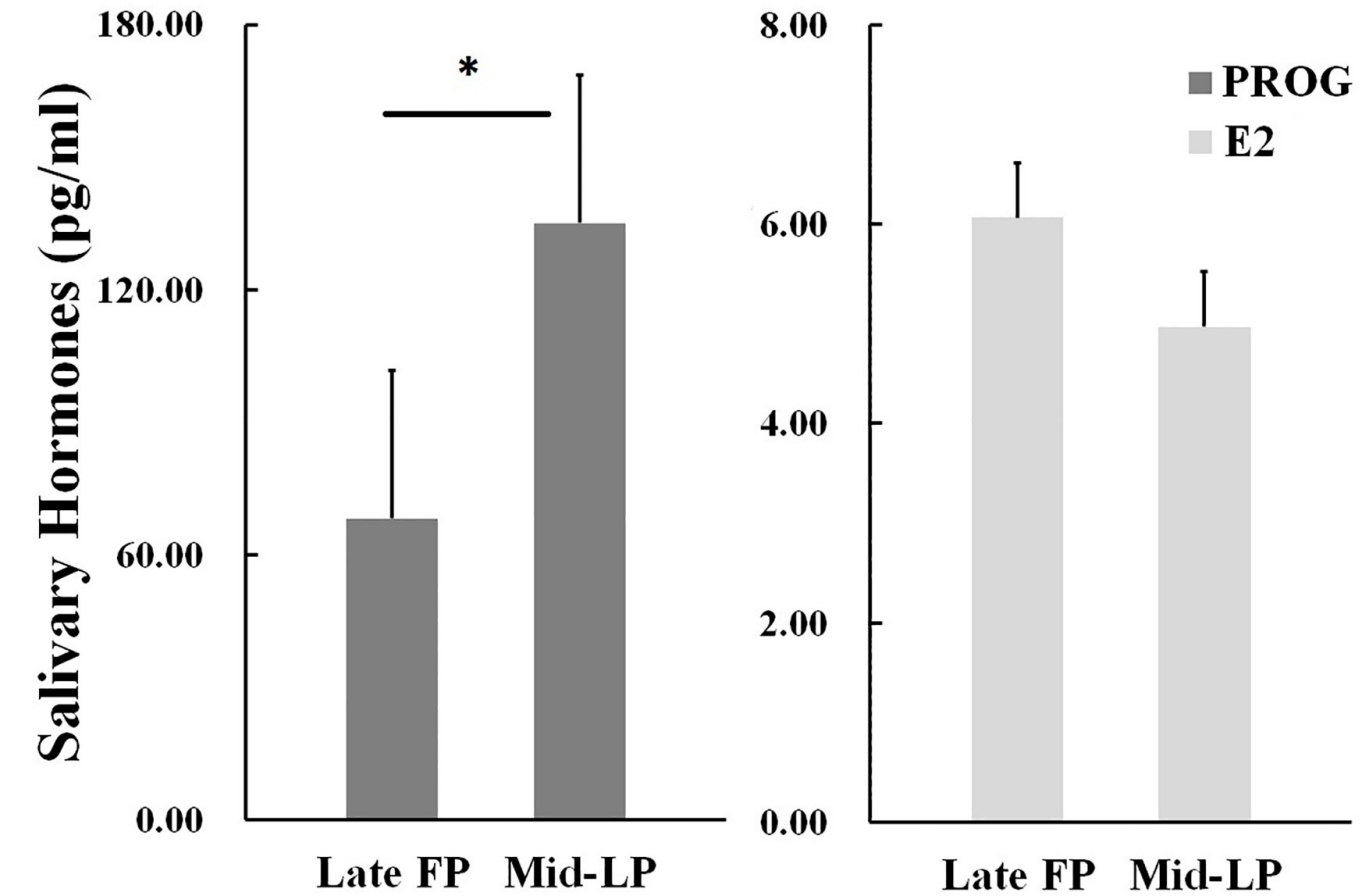
Hormone concentrations for each menstrual phase group. Significant higher PROG levels in the mid-LP group than in the late FP group was indicated. E2 levels were similar between the two groups. (* indicated the significant of effects. Error bars indicate 1 SEM).

Hormone concentrations for each menstrual phase group were reported in Table 1. An independent *t*-test revealed significant higher PROG levels in the mid-LP group than in the late FP group (*t*_2,47_ = 2.029, *p* = 0.048, Cohen’s *d* = 0.592). E2 levels were similar between these two groups (*t*_2,47_ = 1.207, *p* = 0.233, Cohen’s *d* = 0.352, Fig 1). As expected, the late FP and mid-LP had similar E2 levels, but different PROG levels. Furthermore, we found E2 levels were positive correlated with PROG (*r* = 0.336, *p* = 0.018, Table 3), making it also important to disentangle the association between E2 and PROG. Thus, when performed following behavioral and neural correlation analyses which related to ovarian hormones respectively, we set E2 and PROG as each other’s control variable.

**Table 1:** *M* and *SD* of E2 and PROG levels for each menstrual phases.

	late FP (*N* = 25)		mid-LP(*N* = 24)	
	*M*	*SD*	*M*	*SD*
**E2 (pg/ml)**	6.065	3.815	4.968	2.341
**PROG (pg/ml)**	68.172	98.072	135.125	131.148

### PANAS

No difference was observed between the late FP group and mid-LP group in PA (*t*_2,47_ = 0.070, *p* = 0.944, Cohen’s *d* = 0.020; late FP: 2.952 ± 0.712; mid-LP: 2.938 ± 0.738) or NA (*t*_2,47_ = 0.063, *p* = 0.950, Cohen’s *d* = 0.018; late FP: 2.084 ± 0.904; mid-LP: 2.100 ± 0.887).

### BIS/BAS scoring

There was no difference between the late FP group and the mid-LP group on any of the BIS/BAS subscales (BAS-reward responsiveness, *t*_2,47_ = 0.460, *p* = 0.648, Cohen’s *d* = 0.134; BAS drive, *t*_2,47_ = 0.422, *p* = 0.675, Cohen’s *d* = 0.123; BAS-fun seeking, *t*_2,47_ = 0.388, *p* = 0.700, Cohen’s *d* = 0.113; BIS, *t*_2,47_ = 0.329, *p* = 0.744, Cohen’s *d* = 0.096). The *M* and *SD* were presented in Table 2.

**Table 2:** *M* and *SD* of BIS/BAS subscales for each menstrual phases.

	late FP(*N* = 25)		mid-LP(*N* = 24)	
	*M*	*SD*	*M*	*SD*
**BASr**^a^	14.040	1.744	13.833	1.373
**BASd**^b^	12.600	1.756	12.375	1.974
**BASf**^c^	14.920	2.178	15.167	2.278
**BIS**	16.560	2.694	16.333	2.078

^a^BASr = BAS-reward responsiveness.

^b^BASd = BAS drive.

^c^BASf = BAS-fun seeking.

### Correlations between hormone levels and BIS/BAS

There was a trend of negative correlation between PROG levels and BAS drive scores (*r* = -0.267, *p* = 0.061, uncorrected), no other correlations were found between ovarian hormones and BIS/BAS (Table 3).

**Table 3:** BIS/BAS subscales and ovarian hormones correlations (*N* = 49).

	**BASr**^a^	**BASd**^b^	**BASf**^c^	**BIS**	**E2**	**PROG**	**Age**
**BASr**	–	*r* = .494*	*r* = .527*	*r* = .287*	*r* = -.009	*r* = -.071	*r* = .184
		*p* = .000	*p* = .000	*p* = .046	*p* = .953	*p* = .632	*p* = .206
**BASd**	*r* = .494*	–	*r* = .444*	*r* = -.126	*r* = .039	*r* = -.267	*r* = .037
	*p* = .000		*p* = .001	*p* = .388	*p* = .792	*p* = .061	*p* = .803
**BASf**	*r* = .527*	*r* = .444*	–	*r* = .107	*r* = .177	*r* = .036	*r* = .088
	*p* = .000	*p* = .001		*p* = .464	*p* = .229	*p* = .810	*p* = .549
**BIS**	*r* = .287*	*r* = -.126	*r* = .107	–	*r* = .037	*r* = -.126	*r* = -.125
	*p* = .046	*p* = .388	*p* = .464		*p* = .802	*p* = .395	*p* = .392
**E2**	*r* = -.009	*r* = .039	*r* = .177	*r* = .037	–	*r* = .336*	*r* = -.210
	*p* = .953	*p* = .792	*p* = .229	*p* = .802		*p* = .018	*p* = .151
**PROG**	*r* = -.071	*r* = -.267	*r* = .036	*r* = -.126	*r* = .336*	–	*r* = .084
	*p* = .632	*p* = .061	*p* = .810	*p* = .395	*p* = .018		*p* = .568
**Age**	*r* = .184	*r* = .037	*r* = .088	*r* = -.125	*r* = -.210	*r* = .084	–
	*p* = .206	*p* = .803	*p* = .549	*p* = .392	*p* = .151	*p* = .568	

* indicated the significant of effects with uncorrected *p* < 0.05.

^a^BASr = BAS-reward responsiveness.

^b^BASd = BAS drive.

^c^BASf = BAS-fun seeking.

**Fig 2:**
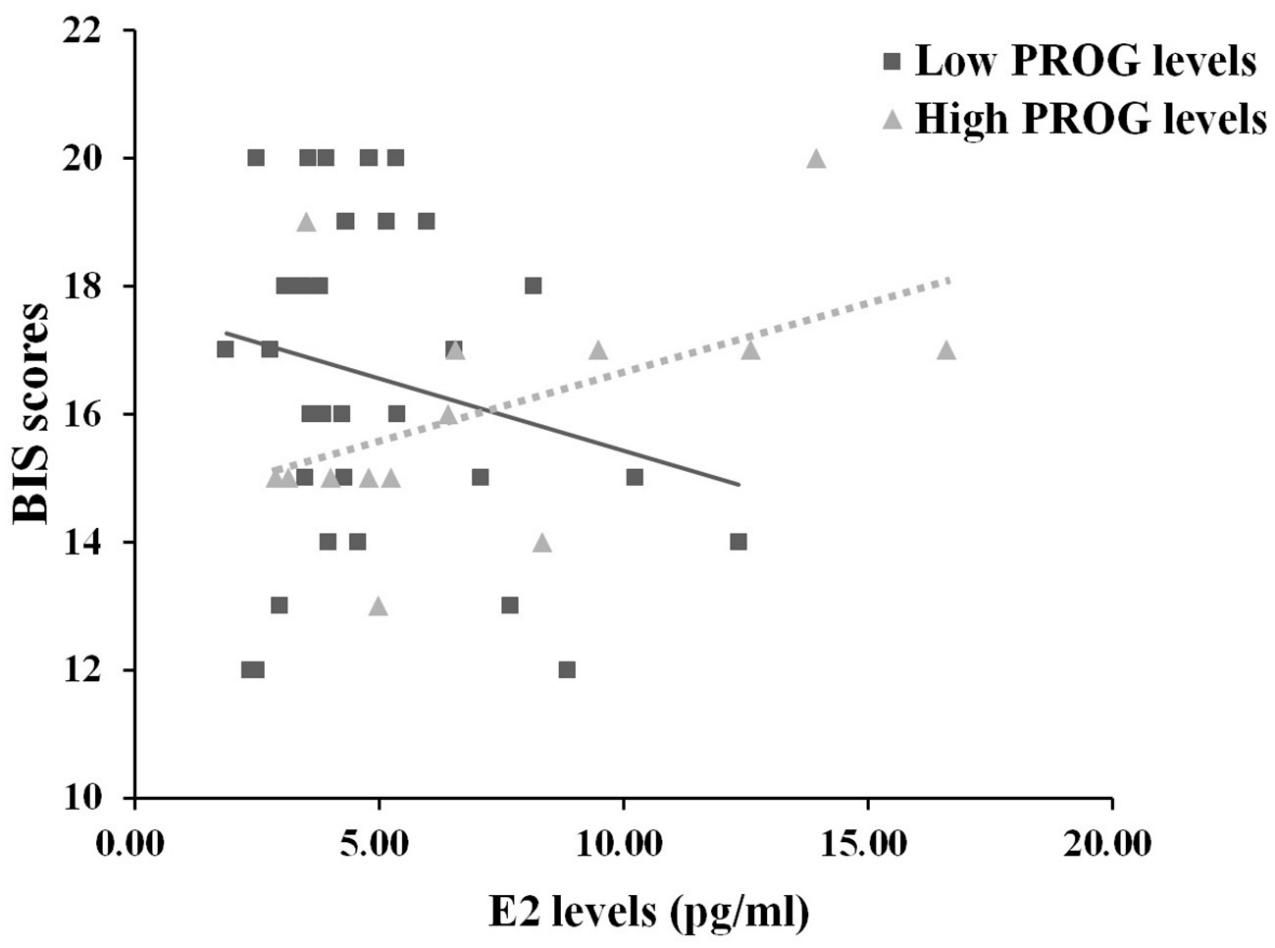
Moderating effects model for BIS and ovarian hormones. When PROG was higher than mean levels, E2 tended to positively correlation with BIS scores; when PROG was lower than mean levels, E2 tended to correlate negatively with the BIS scores.

The moderating analysis revealed a moderating effect of PROG, *F*_1,45_ = 4.494, *p* = 0.040, η_*p*_^*2*^ = 0.557. With higher PROG levels, the conditional effect of E2 on BIS scores tended to be positive (*t*_1,45_ = 1.882, *p* = 0.066), but conditional effect tended to be negative when PROG levels were low (*t*_1,45_ = -1.384, *p* = 0.170), see S1 and S2 Tables in supporting information file. Fig 2 plotted this moderating effect graphically using the coefficients from this model, setting the moderator PROG to its sample mean. No other moderating effects were found.

### ROI based resting-state FC

#### Group effects based on the menstrual cycle

No significant different activation of brain regions were found between late FP and mid-LP groups after FWE correction in the RSFC analyses of VTA or bilateral SN.

#### Ovarian hormones

ROI-to-whole-brain analyses revealed a significant relationship between ovarian hormones levels and RSFC between the VTA/SN and clusters in the frontal and parietal lobe (Table 4). Increased correlated coupling between the SN with inferior parietal lobe was associated with higher PROG levels. On the other side, a significant negative relationship was observed between E2 levels and RSFC between the VTA and the inferior frontal gyrus.

**Table 4:** Correlations between ovarian hormones and brain region activity in whole-brain analysis (*N* = 49).

ROI Seeds	*k*	Corrected *p*-value ^a^	RSFC with	BA	*T*	H	MNI		
							*x*	*y*	*z*
**Positive correlations with PROG levels**									
R SN	83	0.003	Postcentral Gyrus	2	4.42	R	39	-42	69
			Inferior Parietal Lobe	40	4.46	R	36	-42	45
**Negative correlations with E2**									
VTA	71	0.007	Inferior Frontal Gyrus	44	4.55	R	51	12	15
			Superior Temporal Gyrus	44	4.25	R	57	12	33
R SN	94	0.002	Superior Parietal Lobe	7	5.54	L	-21	-72	57
			Inferior Parietal Lobe	7	4.16	L	-21	-63	54
	323	0.000	Inferior Occipital Gyrus	19	4.94	L	-45	-66	-12
			Inferior Temporal Gyrus	37	4.23	L	-48	-60	-6
	147	0.000	Inferior Parietal Lobe	40	4.66	R	36	-42	45
	66	0.011	Inferior Temporal Gyrus	37	4.39	R	57	-51	-12

^a^ Results are reported at Cluster significant after FWE correction at *p* < 0.05 for multiple comparisons; *k* = cluster size, MNI = Montreal Neurological Institute. L, left; R, right; H, hemisphere; BA, broca’s area.

#### BIA/BAS

The BAS drive scores were found significantly correlated with the RSFC strength between VTA and nodes of the reward related brain regions including OFC, anterior cingulate cortex (ACC), medial prefrontal cortex (mPFC), dorsal striatum, and NAc. Meanwhile, negative correlations with the BAS-reward responsiveness were observed between the right SN and the dlPFC. For the BIS, increased correlated coupling between the right SN and dlPFC were associated with higher BIS scores (Table 5).

**Table 5:** Correlations between BIS/BAS and brain region activity in whole-brain analysis (*N* = 49).

ROI Seeds	*k*	Corrected *p*-value ^a^	RSFC with	BA	*T*	H	MNI		
							*x*	*y*	*z*
**Positive correlations with BAS drive scores**									
VTA	114	0.000	Orbitofrontal Cortex	10	4.74	L	-3	51	-6
			Anterior Cingulate Cortex	32	4.33	L	0	42	6
			Medial Superior Frontal Gyrus	10	3.78	L	0	54	6
	52	0.045	Putamen		4.10	R	18	18	-12
			Nucleus accumbens		4.05	R	9	15	-18
			Caudate nucleus		3.97	R	18	27	-6
**Negative correlations with BAS-reward responsiveness**									
R SN	47	0.044	Medial Superior Frontal Gyrus	8	5.76	L	-6	42	54
			Superior Frontal Gyrus	9	3.41	L	-21	36	51
	55	0.022	Cerebellum		5.42	R	6	-51	-48
	47	0.044	Inferior Frontal Gyrus	45	4.93	L	-54	33	6
	87	0.002	Superior Frontal Gyrus	10	4.66	L	-12	57	24
			Medial Superior Frontal Gyrus	10	4.32	L	-15	60	0
**Positive correlations with BIS**									
R SN	54	0.025	Thalamus		5.24	R	21	-15	12
			Putamen		4.02	R	21	-3	12
	53	0.027	Middle Frontal Gyrus	46	4.43	R	45	42	27
			Middle Frontal Gyrus	9	4.01	R	33	45	39

^a^ Results are reported at Cluster significant after FWE correction at *p* < 0.05 for multiple comparisons; *k* = cluster size, MNI = Montreal Neurological Institute. L, left; R, right; H, hemisphere; BA, broca’s area.

**Fig 3:**
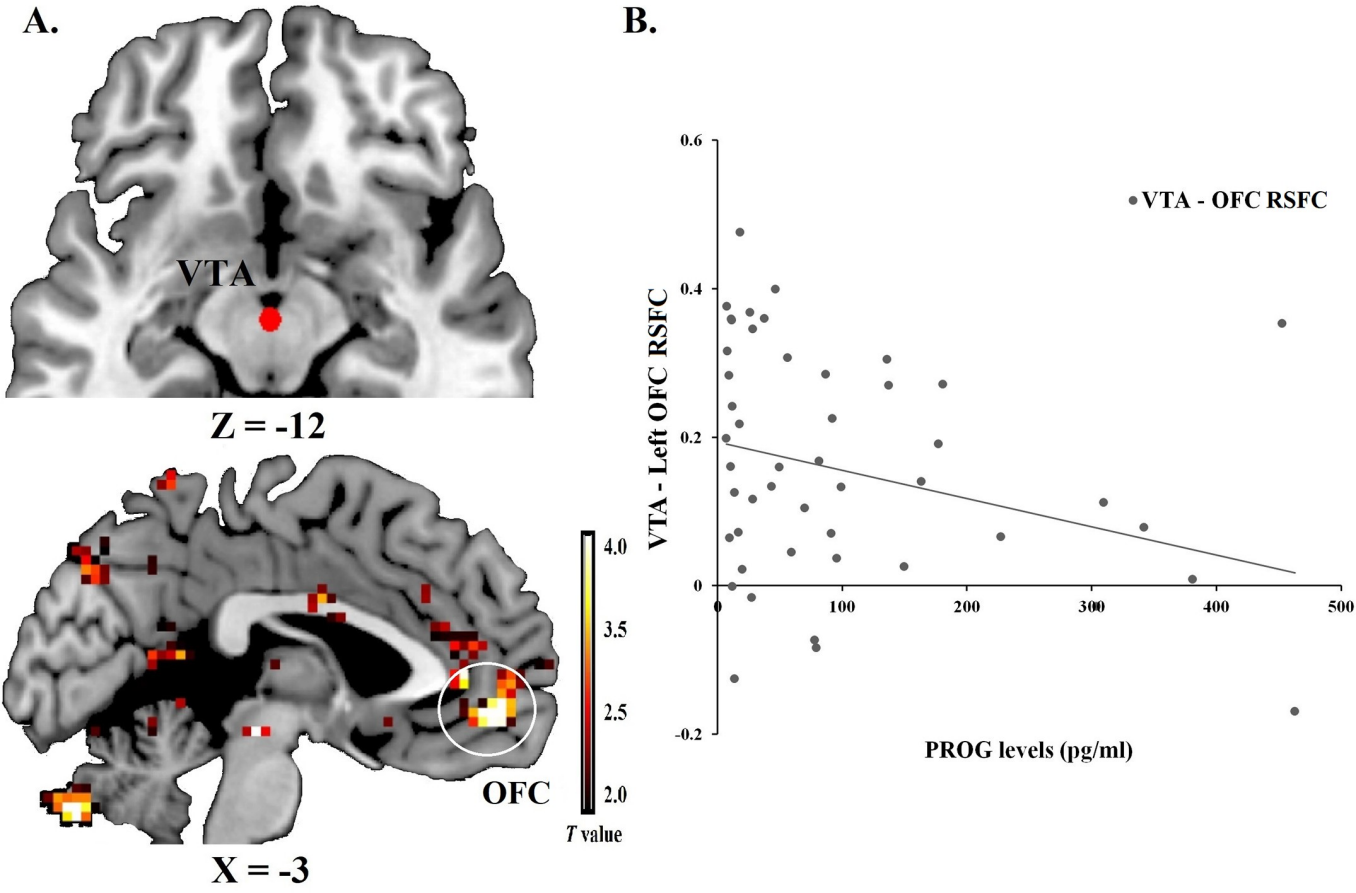
ROI-to-ROI RSFC for the VTA and OFC. (A) ROIs of VTA [MNI: 0, -15, -12] and left OFC [MNI: -3, 51, -6] were displayed. (B) Significantly reduced RSFC was seen for VTA-left OFC ROI pairs when PROG levels were higher.

**Fig 4:**
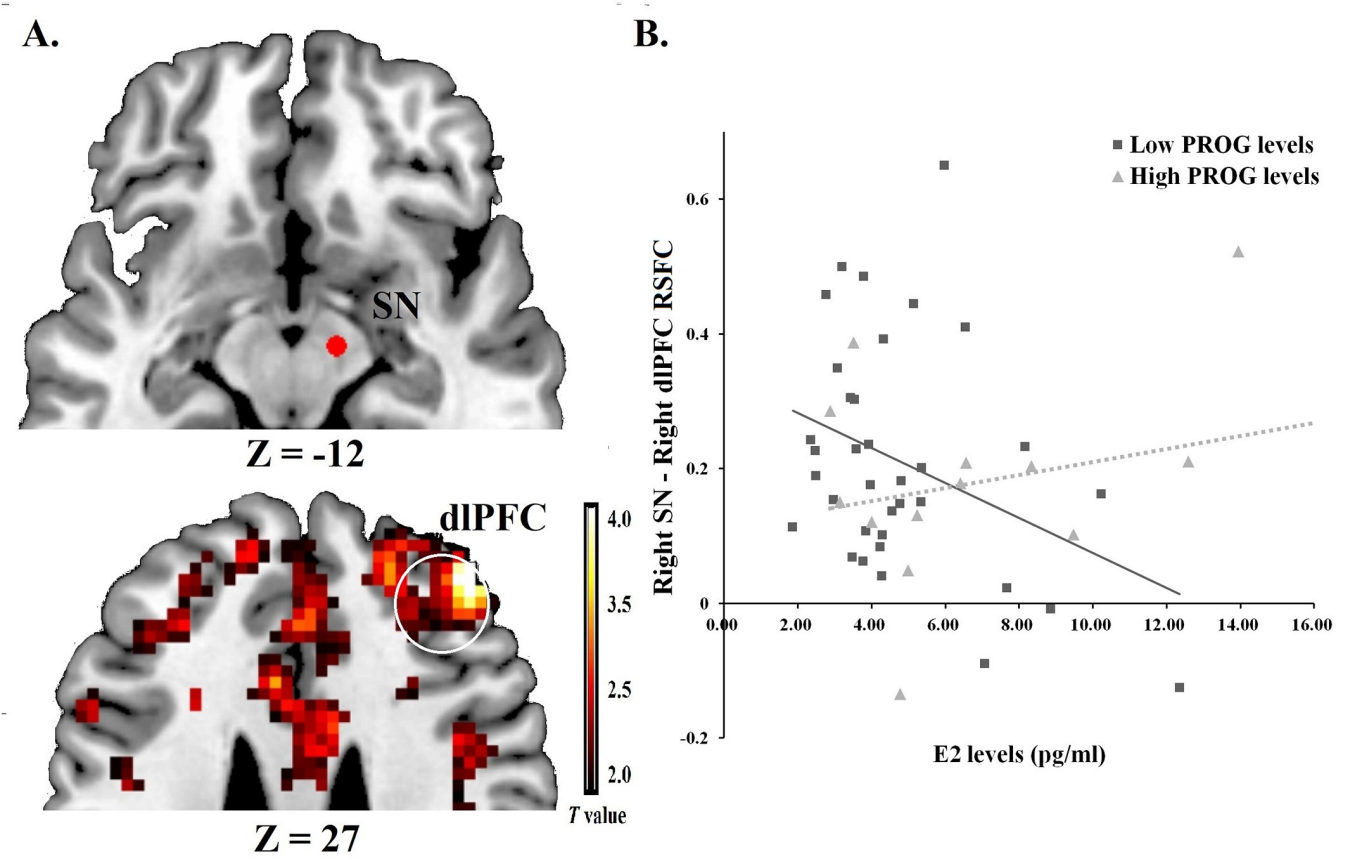
ROI-to-ROI RSFC for the SN and dlPFC. (A) ROIs of right SN [MNI: 12, -12, -12] and right dlPFC [MNI: 45, 42, 27] were displayed. (B) When PROG was higher than mean levels, E2 was positively correlation with the SN-dlPFC RSFC; when PROG was lower than mean levels, E2 correlated negatively with the SN-dlPFC RSFC.

For further examine the relationships between ovarian hormones and the BIS/BAS on the RSFC, we chose the seeds activated in the whole brain analyses to continue computing Fisher’s Z-transformed correlations between BOLD signals of ROIs pairs. We found a negative correlation between PROG levels and the VTA-OFC pair (*r* = -0.283, *p* = 0.049), Fig 3. Moderating effect analysis revealed levels of PROG could moderate relationships between E2 levels and the ROIs pair of SN and dlPFC, *F*_1,45_ = 11.129, *p* = 0.002, η_*p*_^*2*^ = 0.856. Stronger correlated coupling in this pathway was associated with higher E2 levels under high levels of PROG (*t*_1,45_ = 2.414, *p* = 0.020), negative correlation was found with E2 levels under low levels of PROG (*t*_1,45_ = -2.830, *p* = 0.007), see S3 and S4 Tables in supporting information file. Fig 4 plotted this moderating effect with setting the moderator PROG to its sample mean.

## Discussion

Using BIS/BAS scales and the resting-state fMRI, we demonstrated an interaction effect of E2 and PROG on the BIS/BAS and related DA pathways during women’s natural menstrual cycle. Women had high PROG levels in our study, their E2 levels were found positively correlated with BIS scores, but those women who were under low PROG levels condition, their E2 levels were negative correlation with BIS scores. On the brain level, we found BAS was more related with the VTA-NAc pathway, and also included other brain reward regions like OFC. Meanwhile, the BIS was more associated with the SN-dlPFC pathway. ROI-based RSFC analyses further revealed that, the SN-dlPFC pathway could be also modulated by ovarian hormones. With higher PROG levels, increased E2 levels among women were accompanied by stronger RSFC of the SN-dlPFC, but when PROG levels were low, E2 levels were negatively correlated with the SN-dlPFC RSFC. These findings revealed a moderating effect of E2 and PROG on BIS, and the SN-dlPFC pathway was mainly involved in this process.

Previous behavioral and neuroimaging studies suggest that E2 and PROG could have multiple combined influences on reward-related DA pathways and prefrontal areas. Some studies show PROG reduce women’s addiction response to the psychedelic (e.g. cocaine, d-amphetamine) under the facilitating of high E2 levels [25, 26]. Another study on the cognitive function in naturally postmenopausal women indicted that, compared with received E2 or PROG alone, coadministration of E2 and PROG could effectively improve the activation of dlPFC [38]. Recent neurophysiological studies continue to reveal that E2 and PROG exhibit modulatory effects on DA and its metabolites. For example, E2 decreases catechol-O-methyl transferase (COMT) protein expression in the prefrontal cortex of rats [39], while PROG can either up- or down- regulate COMT expression [40]. A microdialysis study found a rapid effect of PROG in striatal DA releasing was enhanced by E2 priming [16]. In the present study, women with high PROG levels showed a positive correlation between E2 and BIS scores, and a similar relationship continued to reveal between E2 and the strength of SN-dlPFC RSFC. However, opposite effects were found between E2 and BIS scores in woman who had low PROG levels, as well a negative correlation was revealed between E2 and the SN-dlPFC RSFC. In the whole-brain RSFC analyses, the SN-dlPFC pathway was found significantly in the positive correlation with BIS scores. Meanwhile, whole-brain analyses also found an opposite RSFC between the VTA/SN and the fronto-pairetal areas when respectively correlated to E2 and PROG levels. Thus, these results supported the combined effects of E2 and PROG on the BIS and related DA pathways, particularly on the SN pathway which was responsible for the inhibition control. Previous works have shown that a change in the self-control ability correlates inversely with changes in E2 levels, decreasing from the low E2 period of the menstrual phase (E2 lowest during menstruation) to the late FP (E2 levels increase gradually in the early FP then rapidly until peaking in the late FP), whereas increasing form the late FP to the mid-LP [20, 41]. The present results further suggest that it is the interaction of E2 and PROG that may modulate women’s inhibition ability.

In addition, we found a negative tendency between PROG levels and BAS drive subscales. On the brain level, PROG was negatively correlated with the ROI-to-ROI RSFC between VTA and OFC. These results revealed that, besides PROG countered E2’s negative association with BIS, it had negative correlation with BAS by itself. The BAS is an approach system that related to the rewards and impulsivity. Past studies have indicated that PROG can decrease reactions to rewards when E2 is low [42]. For example, while intact animals show strong preference for a chamber associated with cocaine, PROG administration diminished the preference for the cocaine-paired chambers in ovariectomized female rats and in male rats [43]. Studies in humans also reported similar findings. When PROG was administered to females with low E2, for instance, it attenuated physiological and subjective (e.g., feeling “high,” “willing to pay”) effects of cocaine [44]. Administration of PROG also reduced urges to smoke cigarettes in female smokers during the early follicular phase [45]. Because those studies addressed the effects of PROG when E2 is low, they suggest that PROG decreases reactions to rewards not only by countering the effects of E2, but also by itself.

Notably, we found VTA pathway, including RSFC with OFC and NAc, was more related to BAS drive scores. These results supported previous studies that BAS subscales showed to correlate with the subcortical striatum and orbitofrontal/ ventromedial prefrontal cortex, both of which were the focus of numerous studies investigating reward coding and association [46]. On the other hand, the SN pathway was positively correlated with the BIS. This result confirmed the hypothesis that the SN-dlPFC pathway was responsible for the BIS. Unlike the VTA pathway, which is more involved in reward sensitivity, the SN pathway has been implicated in cognitive behaviors in rats [47], monkeys [48], and humans [49]. There is evidence that lesions of SN produce prominent alterations in non-motor behavior and profound deficits in working memory because of the SN contains output channels directed at prefrontal areas [50]. Our findings further provided an evidence that the BAS and BIS might have separately neural mechanisms and VTA/SN pathways were responsible respectively for them.

From an evolutionary perspective, researchers proposed that cognitive inhibition mechanisms evolved from a necessity to control social and emotional responses in small groups of hominids for the purposes of cooperation and group cohesion [51]. Some research suggest women’s PROG levels could predict their attention to social stimuli, social threat [52], and desires for social affiliation [53]. Research on both animals and humans also uncover a positive relation between PROG and anxiety [54], the anxiety is considered as an emotional response to keep organisms vigilant to signs of danger [55]. Besides, higher levels of both E2 and PROG have been linked to enhanced executive functions and the activation of prefrontal areas, which could serve the inhibition control [56, 57]. Thus, in the present study, the enhanced BIS, including inhibitory behaviors and negative emotion (e.g. anxiety), and increased activity of the SN-dlPFC pathway with rising PROG levels may fulfill women’s basic needing for reliance on social groups during specific menstrual phase. Even we didn’t found a significant higher BIS scores and more strength RSFC of SN-dlPFC pathway in the mid-LP group than the late FP group, these results still suggested an inner relationship between LP and BIS.

### Limitations, implications and future researches

The current findings that interaction of E2 and PROG on BIS/BAS and DA pathways have important implications for understanding the neural mechanisms that mediate women’s impulsivity and inhibitory behaviors. Excess impulsivity is associated with various neuropsychiatric disorders including obsessive compulsive disorder [58], addiction [59], depression [60], and schizophrenia [61].

There were still several limitations need to be mentioned. First, instead of comparisons between cycle phases, we directly estimating the association between circulating ovarian hormones and BIS/BAS. However, recent research suggests that there is substantial inter-cycle variability in women’s PROG levels [62]. Thus, the between-person differences in PROG we observed may not reflect enduring dispositional differences between women but instead may suggest that women showed more self-inhibition and negative emotion during higher PROG cycles compared to lower PROG cycles. As average cyclical ovarian hormones levels can vary from cycle to cycle within the same women, suggesting both intra- and inter-cycle variation in hormones [63]. It is important to understand how both sources of variance in ovarian hormones—within cycle increases and average cycle levels—are associated with BIS/BAS in the future studies. Second, our interpretations were based on a correlational design, the observed association couldn’t reflect a causal role of ovarian hormones in shifting BIA/BAS. This limitation generally applied to most of the menstrual cycle studies, as these studies followed women at different time points within a given menstrual cycle to examine the correlates of rising hormones levels [52]. Future research could assess women across multiple menstrual cycles to better understand the causal relationships between ovarian hormones, affective states, and self-inhibition control. Last, the lack of direct testing of DA levels across the menstrual phase also weakened our result to claim a direct effects of E2 and PROG on DA pathways. For now, there is no accurate measure of DA levels, but future research could use eye movements to assess the DA levels and to obtain a detailed understanding of the influence of ovarian hormones on BIS/BAS [5].

## Conclusions

In conclusion, ovarian hormones affected women’s BIS/BAS at both the behavioral and neural levels. PROG could modulate the relationship between E2 levels and the BIS. When PROG levels were high, E2 showed positive correlation with the BIS, but when PROG levels were low, E2 levels were negative related with the BIS. The RSFC analyses further support the possibility that high PROG decreased women’s response to BAS on the neural level, and meanwhile, countered E2’s negative association with the SN-dlPFC pathway.

## Supporting information

S1 Fig Maps of ROIs. (A) ROIs of VTA [MNI: 0, -15, -12]; (B) ROIs of right SN [MNI: 12, -12, -12]; (C) ROIs of left SN [MNI: -12, -12, -12].
(TIF)

S2 Fig ROI-to-whole-brain correlation maps between the VTA /SN and ovarian hormones. (A) Maps (sagittal, coronal, axial) of negative correlations between E2 and VTA pathways; (B) Maps (sagittal, coronal, axial) of negative correlations between E2 and right SN pathways; (C) Maps (sagittal, coronal, axial) of positive correlations between PROG and right SN pathways.
(TIF)

S3 Fig ROI-to-whole-brain correlation maps between the VTA /SN and BIS/BAS. (A) Maps (sagittal, coronal, axial) of positive correlations between BAS drive subscales and VTA pathways; (B) Maps (sagittal, coronal, axial) of negative correlations between BAS-reward responsiveness subscales and right SN pathways; (C) Maps (sagittal, coronal, axial) of positive correlations between BIS subscales and right SN pathways.
(TIF)

S1 Table Path coefficients of E2 and moderator PROG for prediction of BIS (*N* = 49). (DOCX)

S2 Table Conditional effect of E2 on BIS at values of the moderator PROG (*N* = 49). (DOCX)

S3 Table Path coefficients of E2 and moderator PROG for prediction of SN-dlPFC strength (*N* = 49). (DOCX)

S4 Table Conditional effect of E2 on SN-dlPFC strength at values of the moderator PROG (*N* = 49). (DOCX)
