# Supplementary figures and images for: How ovarian hormones influence the behavioral activation and inhibition system through the dopamine pathway
Source: PLoS One. 2020 Aug 13;15(8):e0237032. doi: 10.1371/journal.pone.0237032 (PMC7425921; doi:10.1371/journal.pone.0237032)

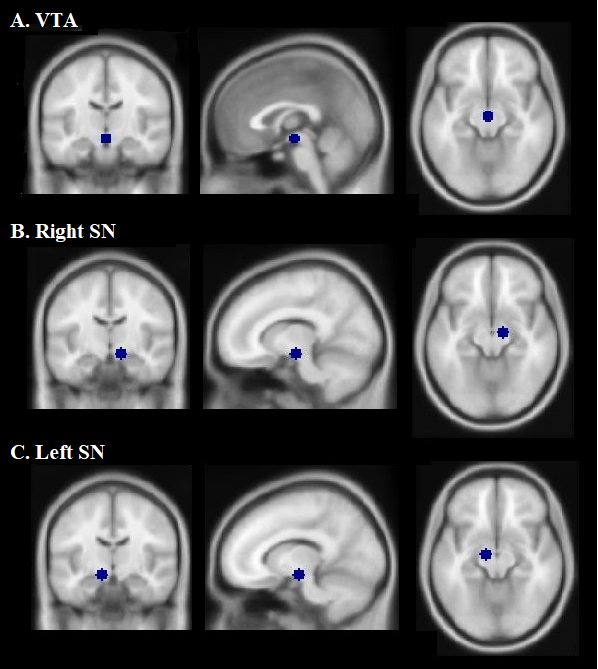

Supplement: S1 Fig — (A) ROIs of VTA [MNI: 0, -15, -12]; (B) ROIs of right SN [MNI: 12, -12, -12]; (C) ROIs of left SN [MNI: -12, -12, -12]. (TIF) [file pone.0237032.s001.tif]

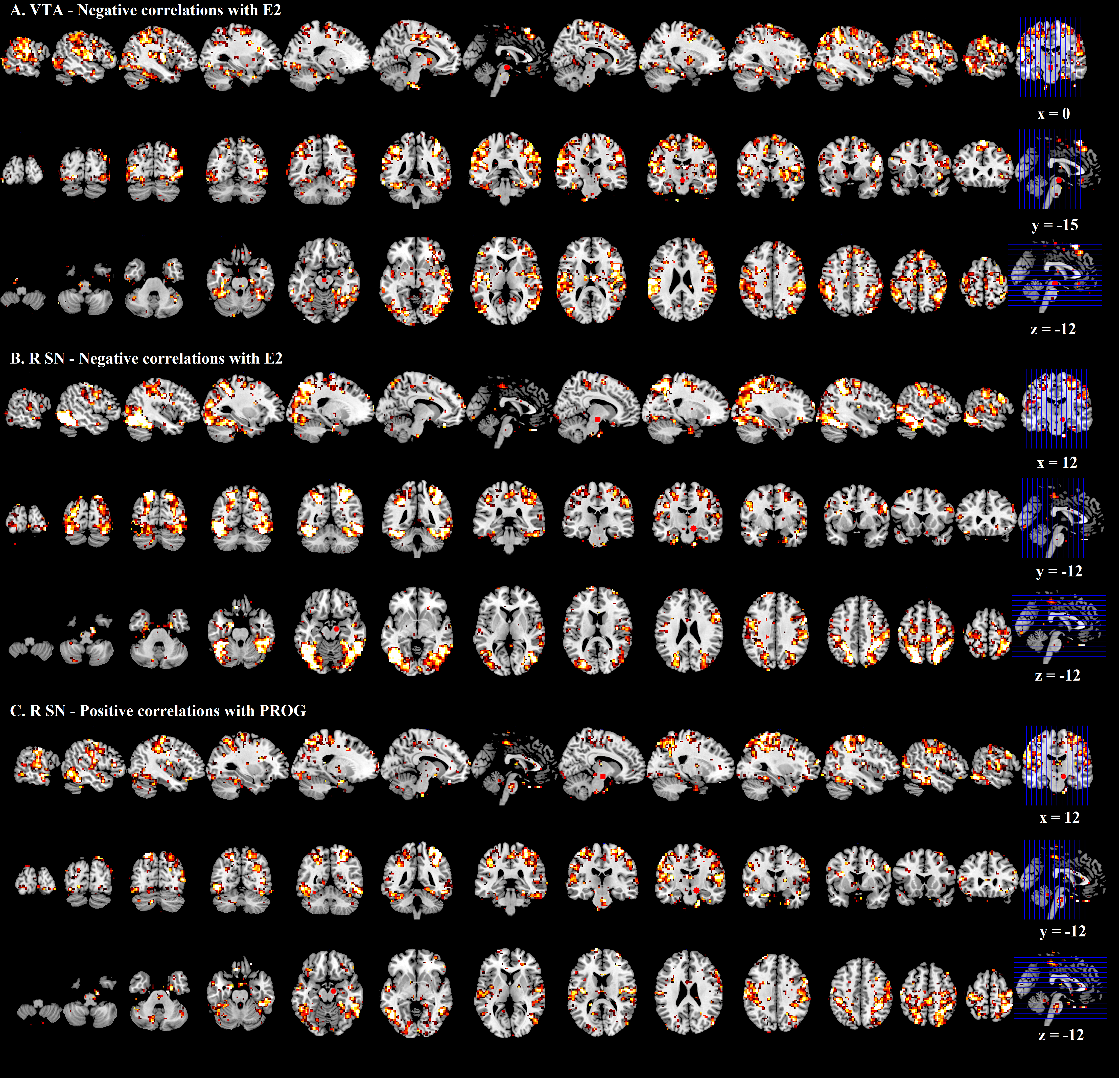

Supplement: S2 Fig — (A) Maps (sagittal, coronal, axial) of negative correlations between E2 and VTA pathways; (B) Maps (sagittal, coronal, axial) of negative correlations between E2 and right SN pathways; (C) Maps (sagittal, coronal, axial) of positive correlations between PROG and right SN pathways. (TIF) [file pone.0237032.s002.tif]

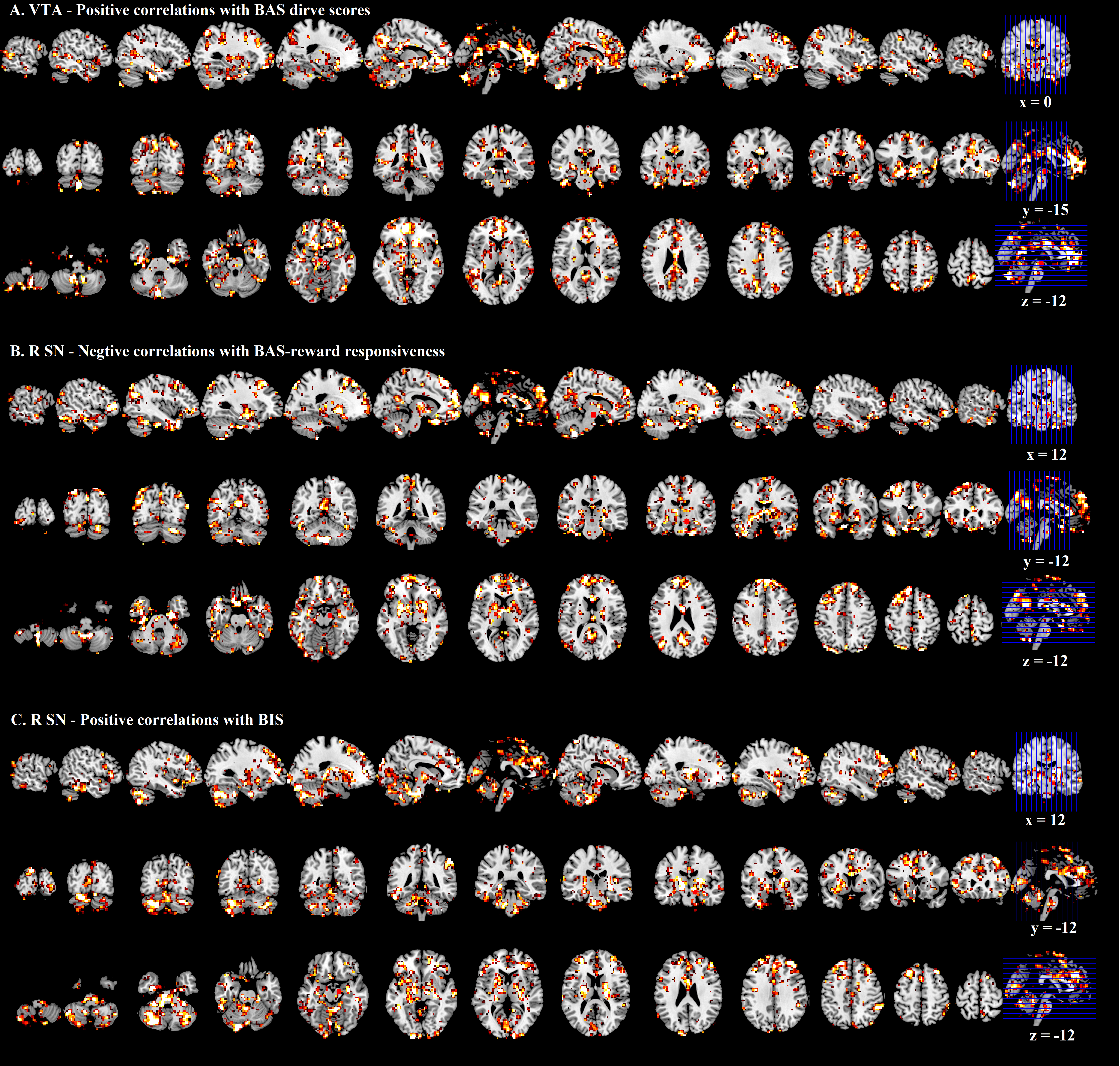

Supplement: S3 Fig — (A) Maps (sagittal, coronal, axial) of positive correlations between BAS drive subscales and VTA pathways; (B) Maps (sagittal, coronal, axial) of negative correlations between BAS-reward responsiveness subscales and right SN pathways; (C) Maps (sagittal, coronal, axial) of positive correlations between BIS subscales and right SN pathways. (TIF) [file pone.0237032.s003.tif]
