# Supplementary material for: How ovarian hormones influence the behavioral activation and inhibition system through the dopamine pathway
Source: PLoS One. 2020 Aug 13;15(8):e0237032. doi: 10.1371/journal.pone.0237032 (PMC7425921; doi:10.1371/journal.pone.0237032)
Supplement: S1 Table — (DOCX) [file pone.0237032.s004.docx]

**S1 Table.**

|  | **Coefficient** | **SE** | ***t/F*** | ***p*** | **LLCI** ^a^ | **ULCI** ^a^ |
| --- | --- | --- | --- | --- | --- | --- |
| **E2** | -0.245 | 0.171 | -1.431 | 0.160 | -0.589 | 0.100 |
| **E2×PROG** | 0.002 | 0.001 | 4.494* | 0.040 | 0.000 | 0.003 |

* indicated the significant of effects with uncorrected *p* < 0.05.

^a^ LLCI = Lower level CI; ULCI = Upper level CI.
