# Supplementary material for: How ovarian hormones influence the behavioral activation and inhibition system through the dopamine pathway
Source: PLoS One. 2020 Aug 13;15(8):e0237032. doi: 10.1371/journal.pone.0237032 (PMC7425921; doi:10.1371/journal.pone.0237032)
Supplement: S2 Table — (DOCX) [file pone.0237032.s005.docx]

**S2 Table.**

| **PROG** | **Effect** | ***SE*** | ***t*** | ***p*** | **LLCI** ^a^ | **ULCI** ^a^ |
| --- | --- | --- | --- | --- | --- | --- |
| **7.210 (-1 *SD*)** | -0.232 | 0.167 | -1.384 | 0.170 | -0.568 | 0.103 |
| **100.965 (*M*)** | -0.068 | 0.121 | -0.565 | 0.575 | -0.312 | 0.176 |
| **220.105 (+1 *SD*)** | 0.139 | 0.124 | 1.132 | 0.264 | -0.109 | 0.389 |
| **339.244(+2 *SD*)** | 0.365 | 0.1940 | 1.882 | 0.066 | -0.026 | 0.756 |

^a^ LLCI = Lower level CI; ULCI = Upper level CI;
