# Supplementary material for: How ovarian hormones influence the behavioral activation and inhibition system through the dopamine pathway
Source: PLoS One. 2020 Aug 13;15(8):e0237032. doi: 10.1371/journal.pone.0237032 (PMC7425921; doi:10.1371/journal.pone.0237032)
Supplement: S4 Table — (DOCX) [file pone.0237032.s007.docx]

**S4 Table.**

| **PROG** | **Effect** | ***SE*** | ***t*** | ***p*** | **LLCI** ^a^ | **ULCI** ^a^ |
| --- | --- | --- | --- | --- | --- | --- |
| **7.210 (-1 *SD*)** | -0.030 | 0.011 | -2.830* | 0.007 | -0.568 | 0.103 |
| **100.965 (*M*)** | -0.014 | 0.008 | -1.765 | 0.084 | -0.312 | 0.176 |
| **220.105 (+1 *SD*)** | 0.001 | 0.008 | 0.922 | 0.362 | -0.109 | 0.389 |
| **339.244(+2 *SD*)** | 0.030 | 0.012 | 2.414* | 0.020 | 0.005 | 0.055 |

* indicated the significant of effects with uncorrected *p* < 0.05.

^a^ LLCI = Lower level CI; ULCI = Upper level CI.
